# Oral ulcer as an exclusive sign of gastric cancer: report of a rare case

**DOI:** 10.1186/1471-2407-5-117

**Published:** 2005-09-19

**Authors:** Piergiuseppe Colombo, Luca Tondulli, Giovanna Masci, Andrea Muzza, Lorenza Rimassa, Duccio Petrella, Armando Santoro

**Affiliations:** 1 Department of Pathology, Istituto Clinico Humanitas, via Manzoni 56, 20089 Rozzano, Milan, Italy; 2 Department of Medical Oncology and Hematology, Istituto Clinico Humanitas, Rozzano, Milan, Italy; 3 Department of Otorynolaringology, Istituto Clinico Humanitas, Rozzano, Milan, Italy

## Abstract

**Background:**

The oral cavity is a rare but occasional target for metastases, which may masquerade as various benign and inflammatory lesions, and sometimes also be asymptomatic. Oral metastatic lesions have been described in various cancers, particularly lung, breast and kidney carcinoma.

**Case presentation:**

We here describe an uncommon case of a hard palate mucosa and gingival metastasis from gastric carcinoma that was originally diagnosed as a periodontal disease.

Histopathological examination of a biopsy of the lesion revealed a signet-ring cell carcinoma, and a subsequent biopsy of an ulcerated stomach lesion showed a poorly differentiated gastric carcinoma. The patient underwent gastric resection but died of heart failure on the tenth postoperative day; a post-mortem examination revealed a residual bilateral ovarian infiltration by gastric carcinoma (Krukenberg's tumor).

**Conclusion:**

An occult carcinoma of the stomach may rarely metastasise to the oral cavity even as a first and exclusive manifestation; it is important to bear this possibility in mind because such conditions may mimic a benign disease.

## Background

Oral metastatic lesions from distant tumours are uncommon, accounting for only 1% of all oral malignancies. They mainly involve the bony structures (particularly the mandible), whereas primary metastases to soft tissues are extraordinarily rare (only 0.1% of oral malignancies) [1]. The most common sites of soft tissue involvement are the gingiva, tongue, lips, and the buccal and palatal mucosa. The primary tumours are mainly lung, breast, kidney and colon tumours, which represent about 70% of the cases reported in the literature. Metastases may produce a variety of signs and symptoms, such as pain, swelling, paresthesia or loose teeth but, in some cases, they are asymptomatic and the lesions are only discovered by chance.

We here describe an extremely rare case of an oral metastasis limited to the hard palate mucosa and gingiva that was originally diagnosed and treated as a periodontal inflammatory disease, but subsequently proved to be the first manifestation of a gastric carcinoma.

## Case presentation

In February 2003, a 61-year-old white female with a history of partial gastrectomy due to a peptic ulcer, and mastectomy due to breast cancer twenty years before, presented with an indolent erythematous mass on her left hard palate and attached gingiva. Her dentist had originally diagnosed periodontal disease, and she was unsuccessfully treated with antibiotics and non-steroidal anti-inflammatory drugs; she subsequently also underwent otolaryngological and radiological examinations of the mouth, but both were negative.

**Figure 1:**
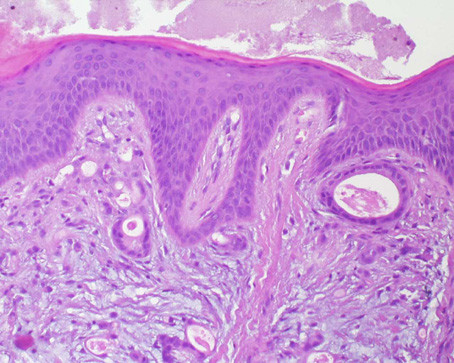
Histopathological findings on hard palate biopsy showing diffuse chorion infiltration by a poorly differentiated carcinoma with a focal microglandular growth pattern (hematoxylin and eosin stain, ×400).

**Figure 2:**
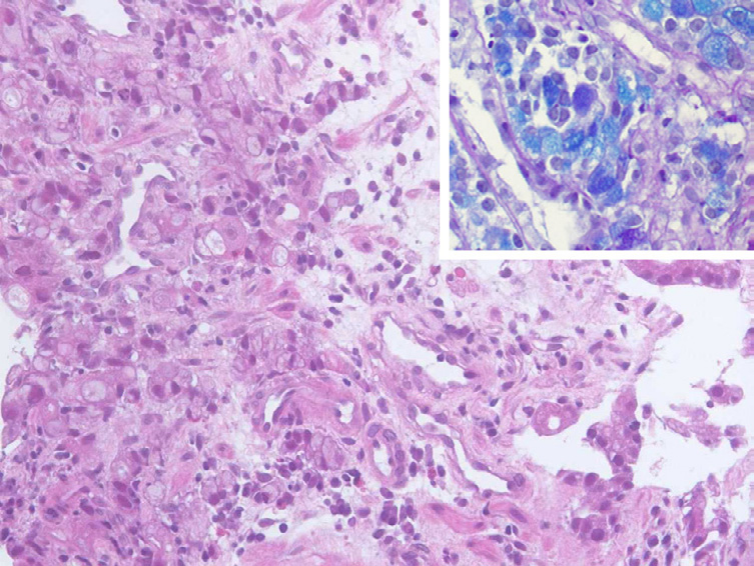
Gastric biopsies revealed an undifferentiated signet-ring cell gastric carcinoma (hematoxylin and eosin stain, ×400). Note the characteristic distribution of blue intracytoplasmic mucines in the neoplastic cells (inset) (Alcian-Pas stain, ×400).

In September 2003, a hard palate biopsy led to a histopathological diagnosis of a chorion metastasis from a breast adenocarcinoma, but the results of subsequent mammography, abdominal ultrasound, chest X-ray and neoplastic marker analyses did not reveal any evidence of a tumour. One month later, a second hard palate biopsy revealed an undifferentiated carcinoma with a focal microglandular and diffuse signet-ring cell growth infiltrating the subepithelial chorion mucosa consistent with a gastric origin (Fig. 1). Although the patient was asymptomatic, gastroscopy showed an ulcerated lesion of the greater curvature of the stomach, and histopathogy revealed an undifferentiated gastric carcinoma with signet-ring cells (Fig. 2).

**Figure 3:**
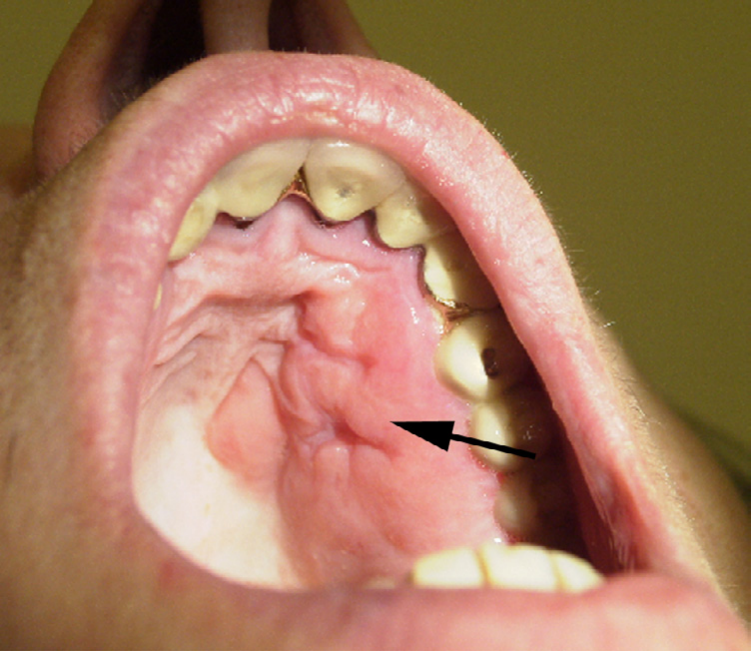
Clinical photograph of the hard palate lesion after biopsy.

**Figure 4:**
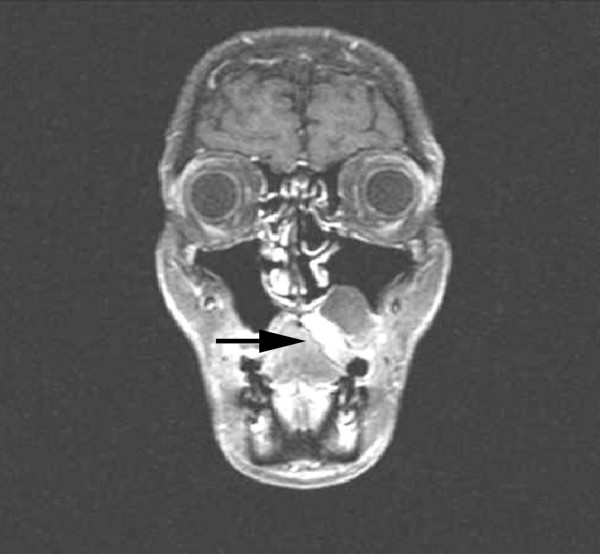
Magnetic resonance image of the maxilla showing an enhanced lesion of the left hard palate with thickening of the genal mucosa and no bone involvement.

She was admitted to our Institution in October 2003, when a physical examination revealed left supraclavicular adenopathy, left exophthalmus, and a firm and painless erythematous mass of nearly 4 cm on her left hard palate, with no evidence of bleeding at palpation (Fig. 3). She was negative for neoplastic markers, and denied any weight loss or gastrointestinal symptoms during the previous weeks. The results of chest and abdominal CT scans were negative, but magnetic resonance imaging of the maxilla revealed an enhanced 2.7 × 2.3 cm lesion on the left hard palate, with thickening of the genal mucosa and no bone involvement (Fig. 4).

A review of the slides of both the oral and gastric lesions confirmed the diagnosis of a mucosal metastasis from a gastric carcinoma, and so the patient underwent five courses of chemotherapy for six months, followed by concomitant 30 Gy radiotherapy of the oral cavity. Because the oral lesion was clinically stable after radiotherapy and was the only sign of distant neoplastic disease, the patient underwent laparotomy with total gastrectomy and regional lymphadenectomy in April 2004. No other macroscopic localizations were found in the abdominal cavity. This curative decision was taken in an attempt to improve the prognosis with the aim of subsequently extirpating the only oral metastasis.

Histopathological examination of the resected specimens revealed a mixed undifferentiated signet-ring cell and microglandular carcinoma infiltrating the gastric wall, with metastases in all of the visceral lymph nodes, and moderate vascular invasion by neoplastic cells. The resected margins were tumour free.

Unfortunately, the patient's condition deteriorated rapidly and she died of heart failure a few days later.

The post-mortem examination demonstrated residual bilateral ovarian infiltration by an undifferentiated signet-ring cell carcinoma (Krukenberg's tumour), with no other abdominal or thoracic localizations.

## Discussion

Although the incidence of gastric carcinoma has markedly decreased over the last twenty years in some countries such as the United States and Great Britain, it remains extremely high in others such as Italy [1,2].

The most frequent sites of metastases from gastric carcinomas are the liver, peritoneum, lung, adrenal gland and ovaries, but there have been some reports of unusual metastases in the uterine body or cervix [3]. Metastatic lesions of gastric origin in the orofacial region are extremely rare, particularly when restricted to oral soft tissues and, in all of the very few cases published in the English literature, the diagnosis of gastric cancer was delayed because of its uncommon clinical presentation (see Table 1) [4-9].

Our case is interesting because the metastatic lesion of the palate was diagnosed before the discovery of the primary tumour, whereas most other previously published reports involve gastric cancers that were already known or simultaneously discovered at the time of the manifestation of the oral lesion [4,5,8,9]. Only Lopez and Arjona have described a case like ours in which the patient developed a gingival lesion before any clinical or pathological evidence of the primary tumour was found [6,7].

All of the patients died as a result of the disease and heart failure a few days later.

In our case, seven months elapsed between the detection of the oral metastasis and the establishment of its gastric origin, possibly because of an initially incorrect interpretation of the clinical history and histopathological features.

From a pathological point of view, both the primary and metastatic lesions had identical features: an undifferentiated pattern, with signet-ring cells mixed with moderately differentiated microglandular neoplastic structures. The oral cavity tumour was restricted to the gingival mucosa (Fig. 4). In this regard, previous investigators have noted that metastatic deposits in the oral soft tissue are most frequently located in the gingiva, followed by the tongue, lips and buccal mucosa [10,11].

It is clinically interesting to note that the oral metastasis was the first indication of an occult primary malignancy in nearly one-third of these patients [11].

In line with the report by Shimoyama *et al*. indicating that the route of metastasis might be hematogenous [9], our case also showed moderate venous invasion by gastric carcinoma cells in the resected stomach, which could explain the oral lesion. In this regard, we agree with other authors who consider patients with oral metastases as having a poor prognosis because of aggressive disease, but it is unclear whether our patient's disease was really widespread at the time of its first manifestation in the gingiva [9-11].

The discovery of an oral metastasis sometimes leads to the detection of an occult malignancy in other body sites, and so it is extremely important to identify it correctly, first clinically and then pathologically.

We stress the peculiarity of hard palate mucosa and gingival involvement from a gastric signet-ring cell carcinoma because metastases from remote organ malignancies in this region may masquerade as various benign and inflammatory lesions [12]. Oral soft tissue and hard palate metastases should therefore be included in the differential diagnosis of ulcerative oral cavity lesions because they may be the first sign of an undiscovered malignancy, and can be easily mistaken for a number of benign diseases.

## Competing interests

The author(s) declare that they have no competing interests.

## Authors' contributions

PC, performed all histopathological examinations, revised the literature, drafted and edited the manuscript. LT, involved in the patient active management, revised the literature, edited the manuscript. GM, LR, AM, involved in the patient active management. DP, performed histopathological figures and carried out the literature search. AS, involved in the final revision of the manuscript and coordinated the submission.

## Pre-publication history

The pre-publication history for this paper can be accessed here:



## Figures and Tables

**Table 1:** Brief summary of clinical and pathological data of English reported cases, and of the current case

Author Year	Age/sex	Site of oral mts	Time relation between primary tumor and mts	Diagnosis of primary tumor	Clinical status of primary tumor	Follow up after diagnosis
Lund et al 1968	63/F	Gingiva	Sincronous	CA undiff.	Unresectable	Died in a few days for respiratory failure
Astacio et al 1969	58/M	Retromolar area	Sincronous	CA grade 3 with signet-ring cells	Resectable	Died in sixth post-oper. day for renal failure
Lopez et al 1976	65/F	Gingiva	3 weeks before primary	CA grade 3	Unresectable	Died in a few days for heart and renal failure
Arjona et al 1989	65/M	Soft palate	5 months before primary	CA nos	Unresectable	Died in a few days for cardio-pulmonary failure
Florio et al 1995	66/M	Hard palate	3 months after primary	CA grade 2	Unresectable	Died in a few days for complications
Shimoyama et al 2004	56/M	Gingiva	15 months after primary	CA grade 3 with signet-ring cells	Resectable	Died two months later for cancer
Current case	61/F	Hard palate and gingiva	7 months before primary	CA undiff. with signet-ring cells	Resectable	Died in tenth post-oper. day for heart failure

mts: metastasis; CA: gastric carcinoma; nos: not otherwise specified
